# Design of a Near Infrared Fluorescent Ureter Imaging Agent for Prevention of Ureter Damage during Abdominal Surgeries

**DOI:** 10.3390/molecules26123739

**Published:** 2021-06-19

**Authors:** Sakkarapalayam M. Mahalingam, Karson S. Putt, Madduri Srinivasarao, Philip S. Low

**Affiliations:** 1 Department of Chemistry, PSG College of Arts and Science and PSG Institute of Advanced Studies, Coimbatore 641004, Tamil Nadu, India; mahalpurdue@gmail.com; 2 Institute for Drug Discovery, Purdue University, West Lafayette, IN 47907, USA; puttk@purdue.edu; 3 Department of Chemistry, Purdue University, West Lafayette, IN 47907, USA; msriniva@purdue.edu

**Keywords:** ureter imaging, fluorescence-guided surgery, near-infrared dye, PEG pharmacokinetics

## Abstract

The inadvertent severing of a ureter during surgery occurs in as many as 4.5% of colorectal surgeries. To help prevent this issue, several near-infrared (NIR) dyes have been developed to assist surgeons with identifying ureter location. However, the majority of these dyes exhibit at least some issue that precludes their widespread usage such as high levels of uptake in other tissues, overlapping emission wavelengths with other NIR dyes used for other fluorescence-guided surgeries, and/or rapid excretion times through the ureters. To overcome these limitations, we have synthesized and characterized the spectral properties and biodistribution of a new series of PEGylated UreterGlow derivatives. The most promising dye, UreterGlow-11 was shown to almost exclusively excrete through the kidneys/ureters with detectable fluorescence observed for at least 12 h. Additionally, while the excitation wavelength is similar to that of other NIR dyes used for cancer resections, the emission is shifted by ~30 nm allowing for discrimination between the different fluorescence-guided surgery probes. In conclusion, these new UreterGlow dyes show promising optical and biodistribution characteristics and are good candidates for translation into the clinic.

## 1. Introduction

Because ureters are not commonly visible on visceral surfaces, their localization during abdominal surgery can be problematic, leading to accidental severance of the ureter in as many as 4.5% of colorectal surgeries [1] and 0.3% of all gynecological procedures [2,3]. To prevent the resulting leakage of urine into the peritoneum and the ensuing long-term complications [1,4,5], any cleaved ureter must be immediately religated by a time-consuming, complicated, and expensive procedure, thereby dramatically increasing the cost and complexity of the surgery. Not surprisingly, considerable effort has been focused on the development of methods to prevent ureter injuries during surgery.

One of the earliest approaches to avoid accidental ureter cleavage was to insert a stent into the ureter that would rigidify the duct and render it detectable by palpation [6,7]. However, because the process of stent insertion was found to cause occasional injury [1,8,9] and since physical palpation was not possible during robotic or endoscopic surgeries, the stent insertion strategy never attracted significant usage. Systemically administered near-infrared (NIR) fluorescent dyes such as indocyanine green (ICG) [10] and UreterGlow [11] were then explored for similar intraoperative ureter visualization, but these initial fluorescent dyes were found to clear primarily through the liver, bile duct, and intestines [10,11], creating high background fluorescence that could mask the location of proximal ureters. While much brighter fluorescent signals have been achieved by intra-ureter dye injection [12,13,14], the injection process has been considered by many surgeons to be too involved for routine ureter localization, leading to similar problems with widespread adoption [15]. Finally, although a zwitterionic near-infrared (NIR) fluorescent dye has been recently designed to excrete predominantly through the ureter [16], its transit time in ureters has been found to be very brief, and its fluorescence excitation and emission unnecessarily overlap with many tumor-targeted NIR dyes (e.g., IR800CW [17,18], LS288 [19,20], ICG [10,12,13,14,15], and OTL38 [11], Table 1), creating a potential discrimination problem when malignant lesions reside near a ureter.

In an effort to create a ureter imaging agent with (1) reduced fluorescence in healthy tissues (except ureters), (2) prolonged transit through the ureters, and (3) an emission spectrum distinct from that of commonly used tumor-targeted fluorescent dyes, we have conjugated a longer wavelength NIR dye to a series of polyethylene glycol (PEG) oligomers of different lengths and examined their clearance following intravenous injection into mice. We report that conjugation of the NIR dye S0456 via a thioether bridge to PEG oligomers of 11–45 oxyethylene units yields ureter imaging agents with little uptake in healthy tissues, prolonged excretion almost exclusively through the ureters, and facile ureter visualization at an emission maximum that is easily distinguished from the common tumor-targeted NIR dyes.

## 2. Methods

### 2.1. Materials

S0456 was purchased from Few Chemicals (Bitterfeld-Wolfen, Germany). 2-(1*H*-7-Azabenzotriazol-1-yl)-1,1,3,3-tetramethyl uronium hexafluorophosphate methanaminium (HATU) was obtained from Genscript Inc. (Piscataway, NJ, USA). The PEG3, PEG11, and PEG2000 oligomers were purchased from TCI America (Portland, OR, USA), BroadPharm (San Diego, CA, USA), and Laysan Bio (Arab, AL, USA), respectively. Diisopropylethylamine (DIPEA), dimethyl sulfoxide (DMSO), and all other chemical reagents were purchased from Sigma-Aldrich (St. Louis, MO, USA). Tubes, pipette tips, microtiter plates, and all other consumables were purchased from Fisher Scientific (Waltham, MA, USA).

### 2.2. Synthesis of PEGylated S0456 NIR Dyes

The S0456 near-infrared dye (1 equiv) was reacted with 2-(4-mercaptophenyl) acetic acid (1.1 equiv) in 2 mL DMSO for 12 h (90% yield, 95% purity). The resulting compound (1 equiv) was coupled to one of three different PEG linkers (1.1 equiv) of sizes 3, 11, and ~45 (PEG2000) in the presence of HATU (1.1 equiv) and DIPEA (5 equiv) in 5 mL DMSO for 6 h to yield the final PEGylated S0456 dyes.

The crude product was purified by preparative reverse-phase high-performance liquid chromatography using a mobile phase of 20 mM ammonium acetate buffer and gradient of 5 to 80% acetonitrile over 30 min (xTerra C18; Waters; 10 μm; 19 × 250 mm). Elution of the conjugate was monitored at 280 nm, and identities of eluted compounds were analyzed by liquid chromatography-mass spectrometry (LC-MS) (see Supplementary Materials). The molecular weight of UreterGlow-0 was calculated (calcd) for [M + H]^+^ (C_46_H_54_N_2_O_14_S_5_): 1019.2, found 1019.3. UreterGlow-3 calcd. (C_54_H_72_N_4_O_16_S_5_): 1193.4, found 1193.6. UreterGlow-11 calcd (C_54_H_72_N_4_O_16_S5): 1545.9, found 1545.9. UreterGlow-45 was synthesized using commercially available PEG2000 which is comprised of PEG oligomers of various lengths that should be centered around a PEG chain length of 45. Calcd. for PEG45 length (C_138_H_240_N_4_O_58_S_5_): 3043.7, found 3001.3, which corresponds to the most prominent PEG chain length centered around 44.

### 2.3. Characterization of Spectral Properties

The excitation and emission wavelengths of the various dyes (1 µM in PBS) were scanned using a Cary Eclipse fluorimeter (Agilent, Santa Clara, CA, USA). The wavelength which resulted in the maximum excitation and emission values for each dye was determined.

### 2.4. Animal Husbandry

ND4 Swiss Webster mice (Harlan Laboratories, Indianapolis, IN, USA) were maintained on normal rodent chow and housed in a sterile environment on a standard 12 h light and dark cycle for the duration of the study. All animal procedures were approved by the Purdue Animal Care and Use Committee in accordance with NIH guidelines (protocol #1111000316 approved 2 February 2021).

### 2.5. In Vivo Biodistribution

Mice were injected via tail vein with 10 nmol of a fluorescent dye conjugate. Mice were sacrificed at 2, 4, and 6 h post-injection, and organs were removed (*n* = 1 per timepoint per conjugate for the initial UreterGlow-0, -3, -11, and -45 biodistribution studies and *n* = 3 per timepoint per conjugate for the UreterGlow-11, IR800CW, IR800BK, and ZW800-1 biodistribution studies). For urine analysis, mice were administered 10 nmol UreterGlow-11 via tail vein injection, sacrificed at various timepoints, and urine was removed from the bladder via syringe. The organs and urine were imaged using a Caliper IVIS Lumina II Imaging Station (PerkinElmer, Waltham, MA, USA) coupled with an ISOON5160 Andor Nikon camera equipped with Living Image Software Version 4.0 (PerkinElmer, Waltham, MA, USA). The settings were as follows: lamp level, high; excitation, 745 nm; emission, ICG; epi illumination; binning (M) 4; FOV, 12.5; f-stop, 4; acquisition time, 1 s.

### 2.6. Effect of pH on UreterGlow-11 Emission Spectra

UreterGlow-11 (1 µM) was added to PBS (pH 7.4), freshly collected human urine (pH 5.5) or sodium carbonate buffered saline with pH values ranging from 2.5 to 10. The dye was excited using 800 nm light and the emission spectrum was obtained using a Cary Eclipse fluorimeter (Agilent, Santa Clara, CA, USA).

## 3. Results and Discussion

### 3.1. Design and Synthesis of the Ureter Probes

**Scheme 1:**
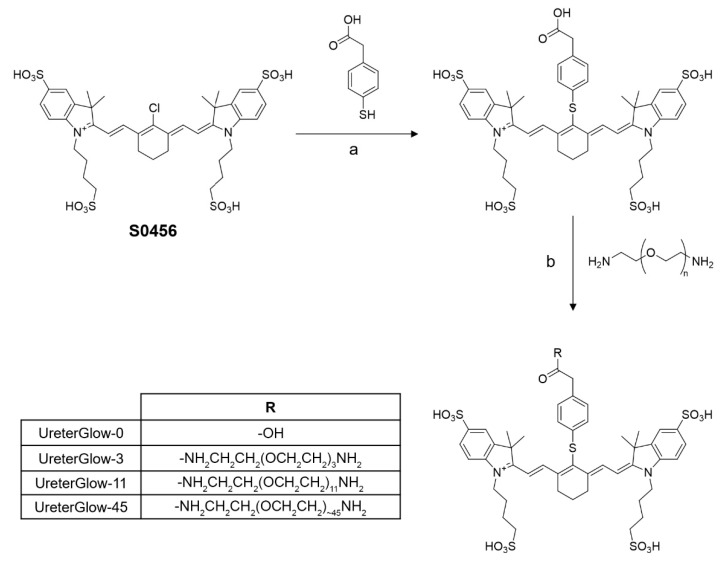
Synthesis of PEGylated NIR dyes for improving renal clearance. (**a**) DMSO, RT, 12 h. (**b**) HATU (1.1 equiv), DIPEA (5 equiv), DMSO, RT, 6 h.

In an effort to remedy the deficiencies of current ureter imaging agents, we undertook to design a water-soluble NIR dye that would (i) excrete for several hours primarily through the ureters, (ii) avoid uptake by normal tissues, (iii) excite with the same light source used for visualization of tumor-targeted fluorescent dyes, and (iv) emit at a longer wavelength than the tumor-targeted fluorescent dyes; i.e., to allow discrimination of the tumor from ureter fluorescence. Because PEGylation can prolong circulation times and reduce nonspecific uptake by healthy tissues [21,22,23], we synthesized a series of optical probes comprised of PEG oligomers of different lengths linked to the cyanine dye, S0456, via a thioether bond to 4-mercaptophenylacetic acid (see Methods). As summarized in Scheme 1, PEG oligomers containing 0, 3, 11, and ~45 oxyethylene units were conjugated to the modified S0456 dye and designated as UreterGlow-0, -3, -11, -45. All four conjugates were purified using preparative reverse-phase HPLC and then characterized by LC-MS (see Supplementary Materials). With sufficient quantities synthesized and purities of >95% achieved for all conjugates, characterization of their physical and biological properties could commence.

### 3.2. Characterization of Physical Properties

**Figure 1:**
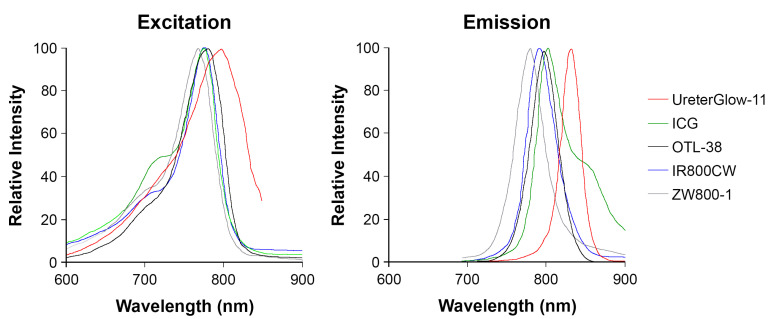
Excitation and emission spectra of various NIR dyes for fluorescence-guided surgery applications. Dyes (1 µM in PBS, pH 7.4) were excited at 680 nm and their emissions were scanned from 700 to 900 nm. Alternatively, excitation wavelengths were scanned from 600 nm to 850 or 900 nm while the emission wavelength was set at 900 nm followed by normalization of their intensities.

Following synthesis of the desired conjugates, molecular weights were confirmed by mass spectrometry, and excitation and emission spectra were obtained using a fluorescence spectrophotometer. As shown in Table 1 and Figure 1, use of a thioether in these UreterGlow conjugates instead of an oxoether bridge connecting the S0456 dye to a phenyl ring shifted the excitation maximum of the conjugate from 776 nm (i.e., similar to OTL38 and many other tumor-targeted fluorescent dyes) to 800 nm. The corresponding emission maxima also shifted from 796 nm to 830 nm, respectively. Because the excitation spectra of both the oxo- and thioether bridged S0456 dyes, as well as the other major NIR dyes used for tumor imaging (IR800CW, LS288, ZW800-1, ICG, and OTL38) overlap over most of their excitation spectra (Figure 1), all of the above NIR dyes should be excitable with the same light source; i.e., avoiding the need to change light sources or cameras to image cancer tissues and ureters simultaneously. Moreover, because the emission spectra of the thioether dyes are shifted ~30–40 nm to longer wavelengths from the major tumor-imaging dyes (Table 1 and Figure 1), it should be possible to display tumor tissue and ureters in different colors on any imaging monitor [24]. Based on these considerations, we expect our ureter probes to function well in combination with most tumor-targeted fluorescent dyes to help prevent accidental ureter damage during abdominal surgeries.

### 3.3. In Vivo Imaging and Biodistribution

**Figure 2:**
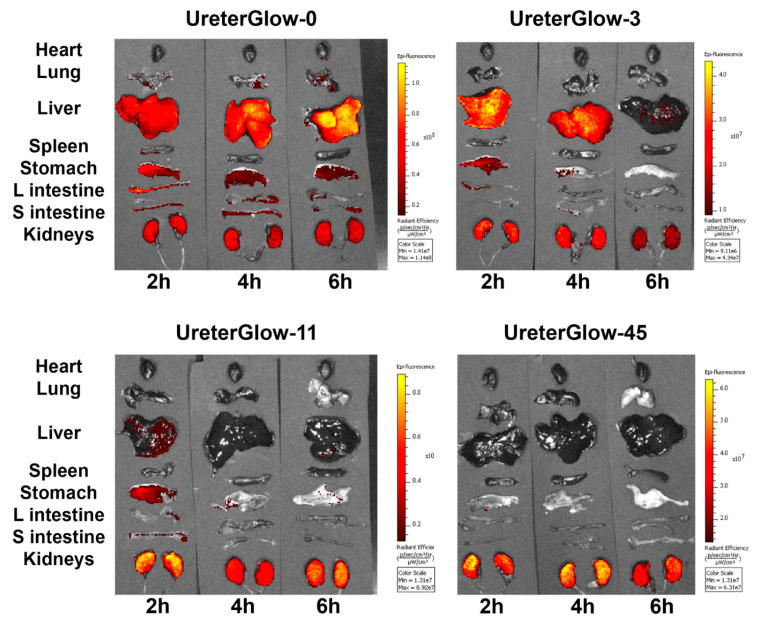
In vivo biodistribution of UreterGlow conjugates. Mice were administered 10 nmol of various conjugates (*n* = 1 per time point per conjugate) via tail vein injection. After varying times, mice were euthanized and their organs were removed. Organs were imaged and the fluorescence intensity recorded.

**Figure 3:**
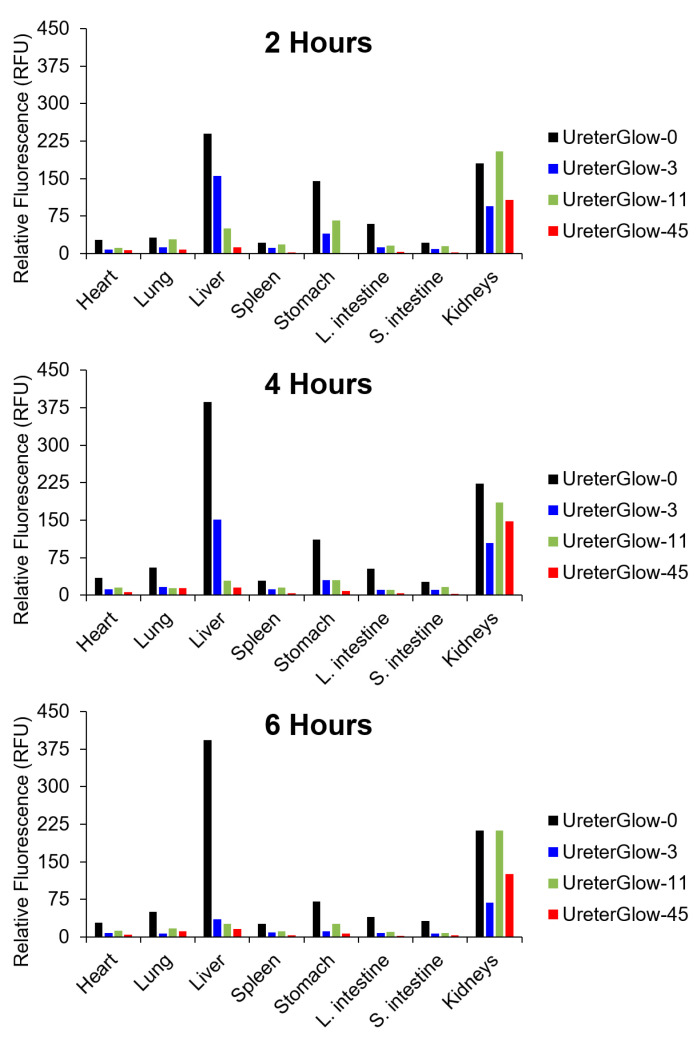
Quantitation of in vivo biodistribution of UreterGlow conjugates. Mice were administered 10 nmol of various conjugates (*n* = 1 per time point per conjugate) via tail vein injection. After varying times, mice were euthanized and their organs were removed. Organs were imaged, fluorescence intensity was recorded, and relative fluorescence was plotted.

To test the hypothesis that a PEG linker of the appropriate length can reduce healthy tissue uptake while prolonging passage of the conjugate through the ureter, the aforementioned PEGylated probes were injected via tail vein into live mice and allowed to circulate for different lengths of time (i.e., 2, 4, and 6 h) before euthanasia and analysis of tissue fluorescence (*n* = 1 for each timepoint and each conjugate). As shown in Figure 2 and Figure 3, UreterGlow-0 showed significant uptake in all major organs except the heart, spleen, and lungs, demonstrating that UreterGlow-0 would not function well for ureter imaging. However, as the length of the appended PEG chain was increased, healthy tissue retention decreased at all time points, with minimal if any healthy tissue fluorescence of UreterGlow-11 and -45 detected at the 2 h time point and no significant healthy tissue fluorescence observed at any subsequent time points. These data demonstrate that the longer PEG chains suppress uptake of the UreterGlow conjugates by healthy tissues, and that their capture by the liver and subsequent excretion via the bile duct into the intestines is also suppressed by longer PEGylation. Because compounds not excreted via the liver/bile duct must excrete through the kidneys, this redirection of UreterGlow-11 and -45 to clearance through the kidneys should enhance and prolong their flow through the ureters. However, because both UreterGlow-11 and -45 performed similarly, UreterGlow-11 was employed in all further studies because it could be synthesized as a homogeneous molecular species.

**Figure 4:**
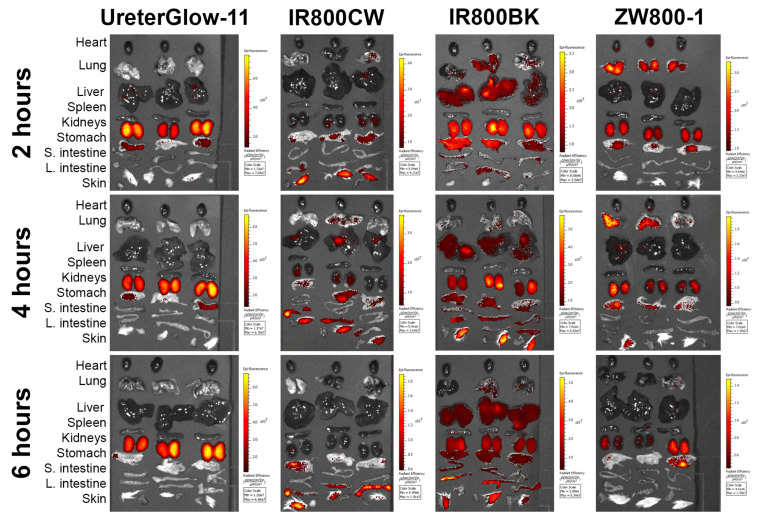
Comparison of in vivo biodistribution of UreterGlow-11 and other NIR ureter imaging dyes/conjugates. Mice were administered 10 nmol of various conjugates (*n* = 3 per time point per conjugate) via tail vein injection. After varying times, mice were euthanized, their organs were removed, fluorescence intensity was imaged.

To compare the properties of UreterGlow-11 with other NIR dyes previously examined for ureter imaging, mice (*n* = 3 per time point per conjugate) were intravenously injected with UreterGlow-11, IR800CW, IR800BK, or ZW800-1) [16,25] and sacrificed 2, 4, or 6 h after injection prior to analysis of tissue-retained fluorescence. As shown in Figure 4, UreterGlow-11 showed little or no uptake in any tissues except the kidneys at all time points examined, suggesting its signal to background contrast along the urinary tract should be very high. In contrast, all other dyes investigated displayed significant accumulation in healthy organs, likely due to their partial excretion through the liver, bile duct and intestines and/or nonspecific retention by an unknown process in these tissues. These nonspecific uptake properties could be troublesome during fluorescence-guided surgeries of metastatic cancers since the latter dyes are also commonly used in fluorescent probes for imaging malignant lesions.

**Figure 5:**
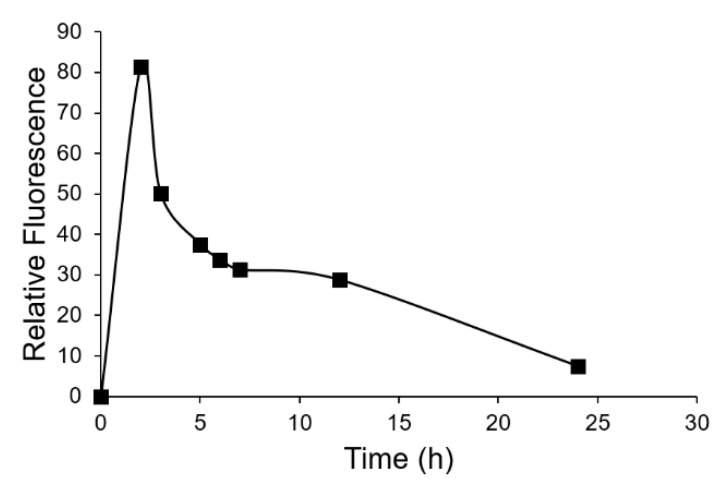
Quantitation of urine fluorescence after administration of UreterGlow-11. Mice were administered 10 nmol of various conjugates (*n* = 1 per time point) via tail vein injection. After varying times, mice were euthanized, and urine was collected from their bladders. The fluorescence of the isolated urine was quantitated.

Although the small size of murine ureters rendered them difficult to image, because any dye that appears in the urine will have recently passed through the ureters, we collected urine samples at different times, post-intravenous injection, and measured their fluorescence intensities in order to confirm that UreterGlow-11 could provide strong ureter fluorescence for prolonged periods following administration. As shown in Figure 5, urine fluorescence remained high for at least 12 h after UreterGlow-11 infusion and then only gradually declined over the subsequent 12 h. These data suggest that UreterGlow-11 should illuminate ureters well, even during protracted abdominal surgeries.

**Figure 6:**
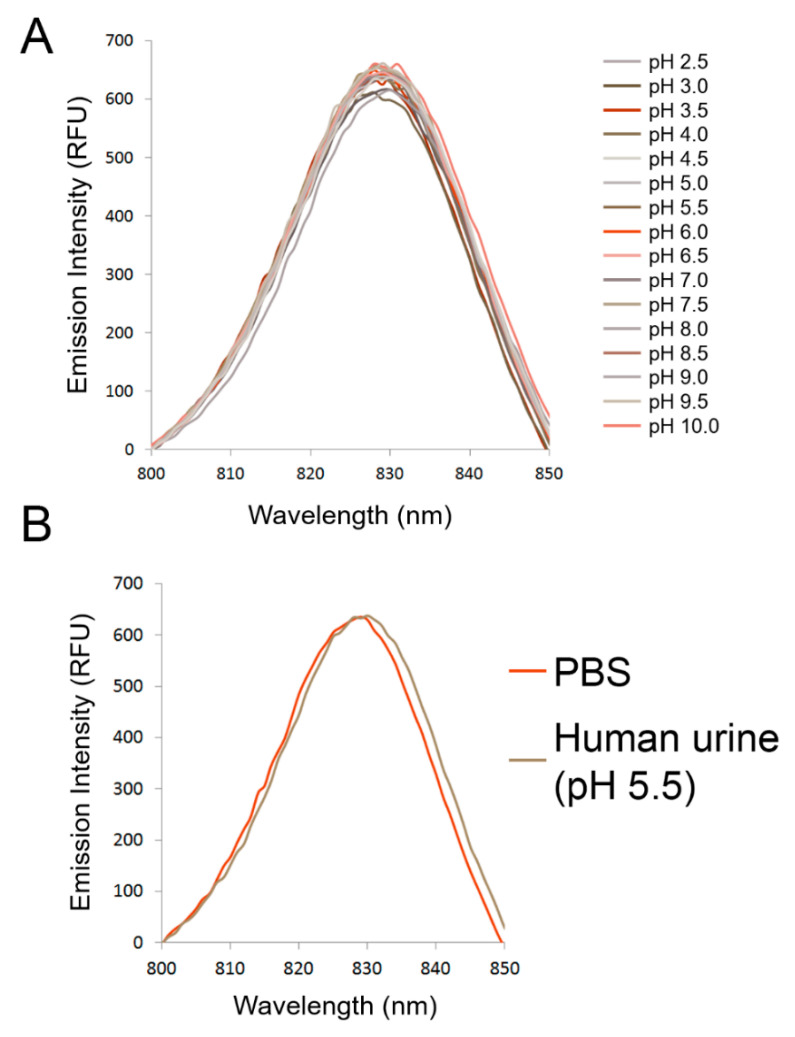
Sensitivity of UreterGlow-45 emission spectra in different pH buffers. (**A**) UreterGlow-11 (1 µM) was excited at 800 nm while dissolved in sodium acetate buffered saline at various pH levels, and the emission spectra were characterized. (**B**) UreterGlow-11 (1 µM) was excited at 800 nm while dissolved in PBS (pH 7.4) or human urine (pH 5.5), and the emission spectra were characterized.

Finally, because urine pH can vary from pH 4.5 to pH 8 [26], it was important to ensure that the UreterGlow-45 fluorescence did not vary with urine pH. As shown in Figure 6A, the emission spectrum of UreterGlow-45 was independent of pH between 2.5 and 10 and also showed no impact when dissolved in urine (Figure 6B). Taken together, these data collectively suggest that UreterGlow-11 should perform well as a ureter imaging agent during abdominal surgeries for cancer.

## 4. Conclusions

Although this brief report described only the impact of two compositional variables on the properties of a NIR dye for intra-operative ureter imaging, many other modifications could also have been explored for further optimization. Thus, NIR dyes with other excitation and emission wavelengths could have been generated by the insertion of other heteroatoms at other locations in the UreterGlow conjugate. PEGs of intermediate lengths between 11 and 45 oxyethylene units could also have been examined for improved biodistribution and pharmacokinetic properties. And finally, a targeting ligand could have been designed that would enable sustained binding of the fluorescent conjugate to the epithelial cells lining the ureters. Thus, while the above improvements in ureter specificity, emission wavelength, and transit time through the ureters now renders UreterGlow-11 a good candidate for translation into the clinic, opportunities may remain for further optimization with an eventual goal of totally eliminating accidental damage to ureters during abdominal surgeries.

## Figures and Tables

**Table 1:** Photo-physical properties of NIR dyes used for fluorescence-guided surgeries and new PEGylated probes.

NIR Dyes	Excitation Maximum (nm)	Emission Maximum (nm)	Stokes Shift (nm)
IR800CW	780	795	15
LS288	770	785	15
ZW800-1	770	788	18
ICG	780	802	22
OTL38	776	796	20
UreterGlow	800	830	30
UreterGlow-0	800	830	30
UreterGlow-3	800	830	30
UreterGlow-11	800	830	30
UreterGlow-45	800	830	30

## Data Availability

The data presented in this study are available in Putt KS. 2021. Supplemental Information Design of a near infrared fluorescent ureter imaging agent for prevention of ureter damage during abdominal surgeries; Zenodo http://doi.org/10.5281/zenodo.4987325.

## Supplementary Materials

The following are available online, Figure S1: Chemical Structures of Selected Dyes Used in Fluorescence-Guided Surgeries; Figure S2: Structure and LC-MS Characterization of UreterGlow-0; Figure S3: Structure and LC-MS Characterization of UreterGlow-3; Figure S4: Structure and LC-MS Characterization of UreterGlow-11; Figure S5: Structure and LC-MS Characterization of UreterGlow-45.

## Sample Availability

Samples of the compounds are not available from the authors.

## References

[1] Silva G., Boutros M., Wexner S. (2012). Role of prophylactic ureteric stents in colorectal surgery. Asian J. Endosc. Surg..

[2] Packiam V.T., Andrew C., Joseph P., Charles N., Sarah F., Gregory B. (2016). The Impact of Minimally Invasive Surgery on Major Iatrogenic Ureteral Injury and Subsequent Ureteral Repair during Hysterectomy: A National Analysis of Risk Factors and Outcomes. Urology.

[3] Barber M.D., Visco A.G., Weidner A.C., Amundsen C.L., Bump R.C. (2000). Bilateral uterosacral ligament vaginal vault suspension with site-specific endopelvic fascia defect repair for treatment of pelvic organ prolapse. Am. J. Obstet. Gynecol..

[4] Sandberg E.M., Cohen S.L., Hurwitz S., Einarsson J.I. (2012). Utility of cystoscopy during hysterectomy. Obstet. Gynecol..

[5] Sharp H.T., Adelman M.R. (2016). Prevention, Recognition, and Management of Urologic Injuries during Gynecologic Surgery. Obstet. Gynecol..

[6] Chou M.T., Wang C.J., Lien R.C. (2009). Prophylactic ureteral catheterization in gynecologic surgery: A 12-year randomized trial in a community hospital. Int. Urogynecol. J. Pelvic Floor Dysfunct..

[7] Speicher P.J., Goldsmith Z.G., Nussbaum D.P., Turley R.S., Peterson A.C., Mantyh C.R. (2014). Ureteral stenting in laparoscopic colorectal surgery. J. Surg. Res..

[8] Dadkhah F., Yari H., Ali Asgari M., Fallahnezhad M.H., Tavoosian A., Ghadian A. (2016). Benefits and Complications of Removing Ureteral Stent Based on the Elapsed Time after Renal Transplantation Surgery. Nephrourol. Mon..

[9] Fanning J., Fenton B., Jean G.M., Chae C. (2011). Cost analysis of prophylactic intraoperative cystoscopic ureteral stents in gynecologic surgery. J. Am. Osteopath. Assoc..

[10] Cherrick G.R., Stein S.W., Leevy C.M., Davidson C.S. (1960). Indocyanine green: Observations on its physical properties, plasma decay, and hepatic extraction. J. Clin. Investig..

[11] Mahalingam S.M., Dip F., Castillo M., Roy M., Wexner S.D., Rosenthal R.J., Low P.S. (2018). Intraoperative Ureter Visualization Using a Novel Near-Infrared Fluorescent Dye. Mol. Pharm..

[12] Siddighi S., Yune J.J., Hardesty J. (2014). Indocyanine green for intraoperative localization of ureter. Am. J. Obstet. Gynecol..

[13] Rajanbabu A., Patel V.J. (2020). Ureteric mapping with Indocyanine green: A new tool to prevent ureteral injury in complex gynecological surgery. J. Endometr. Pelvic Pain Disord..

[14] Lee Z., Moore B., Giusto L., Eun D.D. (2015). Use of indocyanine green during robot-assisted ureteral reconstructions. Eur. Urol..

[15] Cabanes M., Boria F., Gutiérrez A.H., Zapardiel I. (2019). Intra-operative identification of ureters using indocyanine green for gynecological oncology procedures. Int. J. Gynecol. Cancer.

[16] Slooter M., Janssen A., Bemelman W., Tanis P., Hompes R. (2019). Currently available and experimental dyes for intraoperative near-infrared fluorescence imaging of the ureters: A systematic review. Tech. Coloproctol..

[17] Korb M.L., Hartman Y.E., Kovar J., Zinn K.R., Bland K.I., Rosenthal E.L. (2014). Use of monoclonal antibody-IRDye800CW bioconjugates in the resection of breast cancer. J. Surg. Res..

[18] Shrivastava A., Ding H., Kothandaraman S., Wang S.-H., Gong L., Williams M., Milum K., Zhang S., Tweedle M.F. (2014). A High-Affinity Near-Infrared Fluorescent Probe to Target Bombesin Receptors. Mol. Imaging Biol..

[19] Kanduluru A.K., Srinivasarao M., Low P.S. (2016). Design, Synthesis, and Evaluation of a Neurokinin-1 Receptor-Targeted Near-IR Dye for Fluorescence-Guided Surgery of Neuroendocrine Cancers. Bioconjug. Chem..

[20] Wayua C., Low P.S. (2014). Evaluation of a cholecystokinin 2 receptor-targeted near-infrared dye for fluorescence-guided surgery of cancer. Mol. Pharm..

[21] Hamidi M., Rafiei P., Azadi A. (2008). Designing PEGylated therapeutic molecules: Advantages in ADMET properties. Expert Opin. Drug Discov..

[22] Milla P., Dosio F., Cattel L. (2012). PEGylation of proteins and liposomes: A powerful and flexible strategy to improve the drug delivery. Curr. Drug Metab..

[23] Mozar F.S., Chowdhury E.H. (2018). Impact of PEGylated nanoparticles on tumor targeted drug delivery. Curr. Pharm. Des..

[24] De Boer E., Harlaar N.J., Taruttis A., Nagengast W.B., Rosenthal E.L., Ntziachristos V., van Dam G.M. (2015). Optical innovations in surgery. Br. J. Surg..

[25] Van den Bos J., Al-Taher M.A., Bouvy N.D., Stassen L.P.S. (2019). Near-infrared fluorescence laparoscopy of the ureter with three preclinical dyes in a pig model. Surg Endosc..

[26] Bono M.J., Reygaert W.C. (2021). Urinary Tract Infection. StatPearls [Internet].

