# Supplementary material for: Design of a Near Infrared Fluorescent Ureter Imaging Agent for Prevention of Ureter Damage during Abdominal Surgeries
Source: Molecules. 2021 Jun 19;26(12):3739. doi: 10.3390/molecules26123739 (PMC8234099; doi:10.3390/molecules26123739)
Supplement: Supplementary file 1 [file molecules-26-03739-s001.zip › molecules-1259854-supplementary.pdf]

Supplemental Information

Design of a near infrared fluorescent ureter imaging agent for prevention of ureter damage during abdominal surgeries

Sakkarapalayam M Mahalingam[1], Karson S. Putt[2], Madduri Srinivasarao[3], Philip S. Low[2,3,*]

[1] Department of Chemistry, PSG College of Arts and Science and PSG Institute of Advanced Studies, Coimbatore-641004, Tamil Nadu, India
[2] Institute for Drug Discovery, Purdue University, West Lafayette IN 47907 USA
[3] Department of Chemistry, Purdue University, West Lafayette IN 47907 USA

*Author to whom all correspondence should be addressed:

Philip S. Low
Email: plow@purdue.edu
Phone: 765-494-5272

Department of Chemistry
Purdue University
720 Clinic Drive
West Lafayette IN 47907

Table of Contents

Figure S1 – Chemical Structures of Selected Dyes Used in Fluorescence-Guided Surgeries.................... 2
Figure S2 - Structure and LC-MS Characterization of UreterGlow-0 3
Figure S3 - Structure and LC-MS Characterization of UreterGlow-3 4
Figure S4 - Structure and LC-MS Characterization of UreterGlow-11 5
Figure S5 - Structure and LC-MS Characterization of UreterGlow-45 6

Figure S1 - Chemical Structures of Selected Dyes Used in Fluorescence-Guided Surgeries

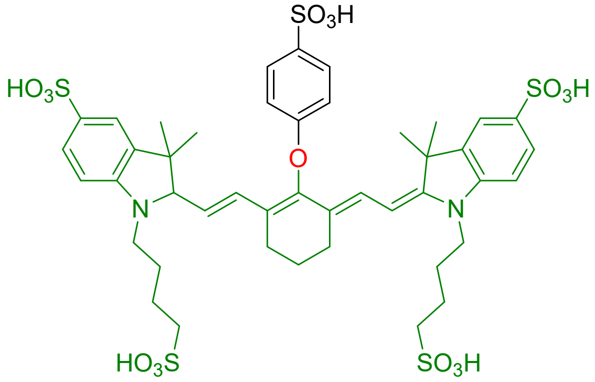

IR800BK

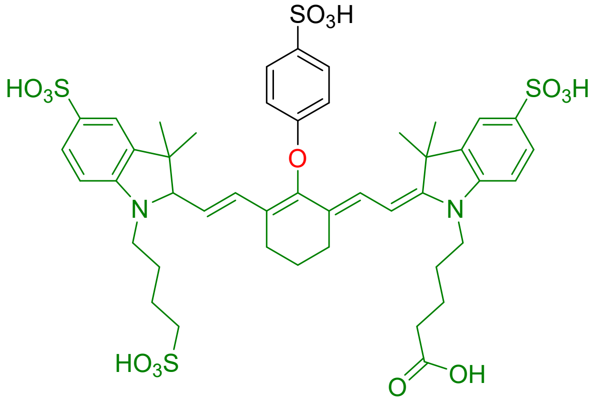

IR800CW

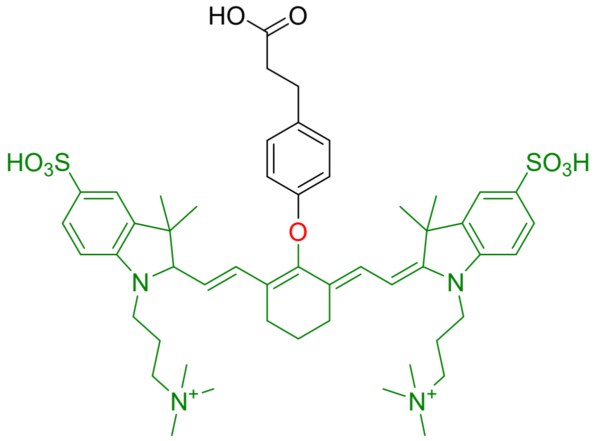

ZW-800-1

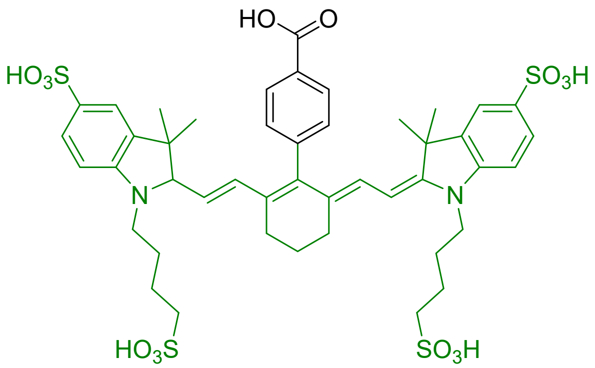

LS288

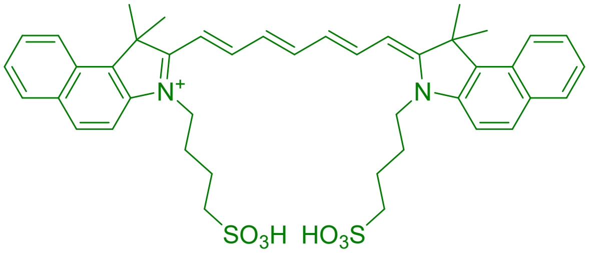

Indocyanine Green (ICG)

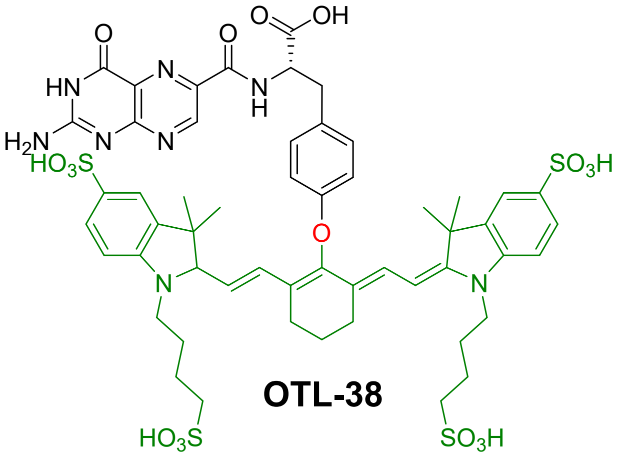

OTL-38

Figure S2 - Structure and LC-MS Characterization of UreterGlow-0

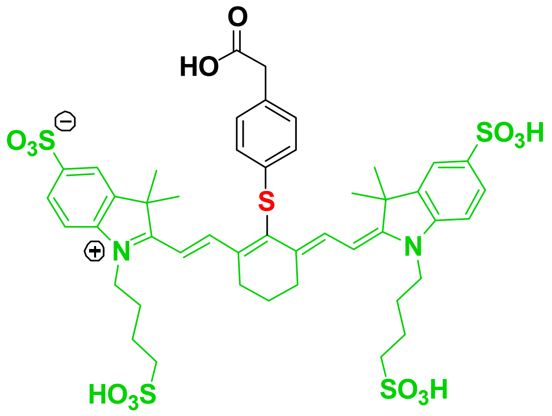

Chemical Formula: $C_{46}H_{54}N_2O_{14}S_5$
Molecular Weight: 1019.23800
UreterGlow-0

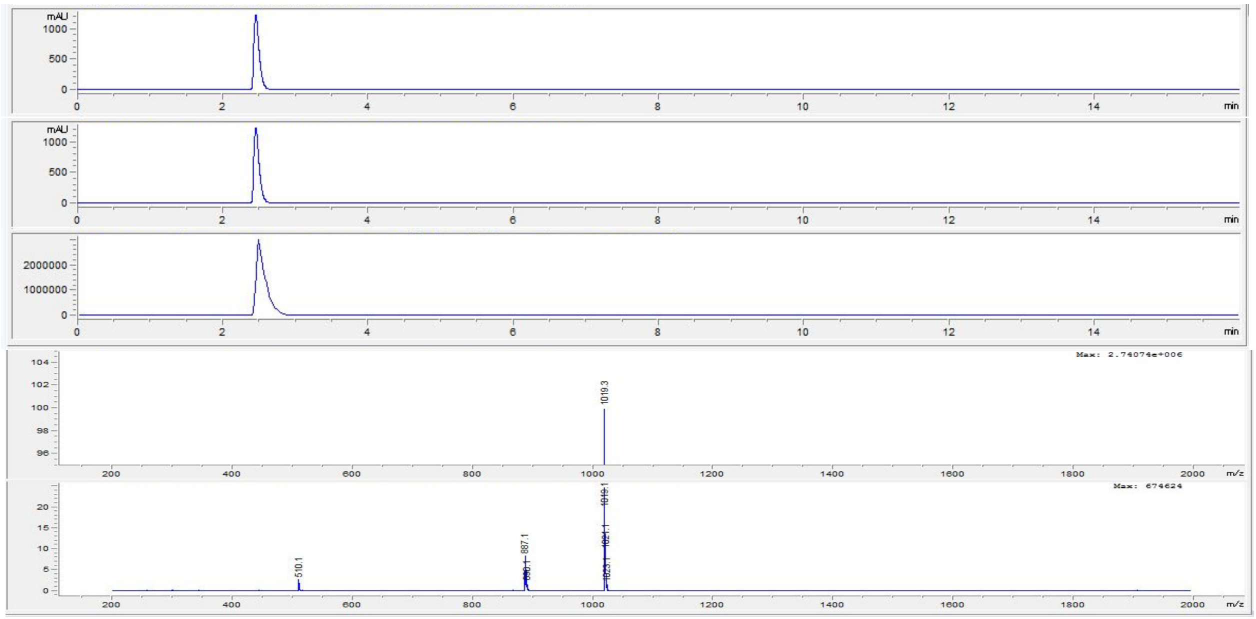

Chromatographic spectra at 280, 260, and 210 nm. Mass spectra at specific mass and total ion spectra.

Figure S3 - Structure and LC-MS Characterization of UreterGlow-3

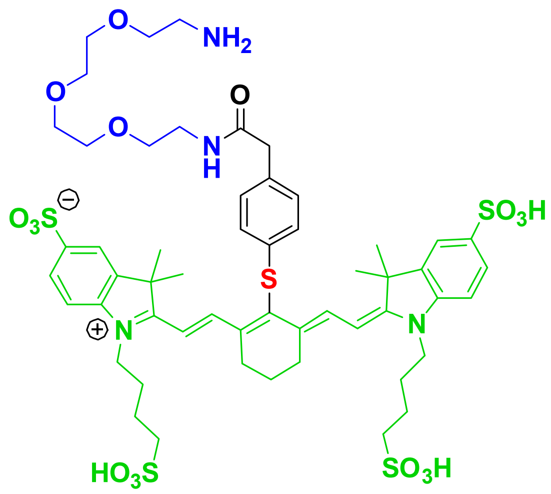

Chemical Formula: $C_{54}H_{72}N_4O_{16}S_5$
Molecular Weight: 1193.48200
UreterGlow-3

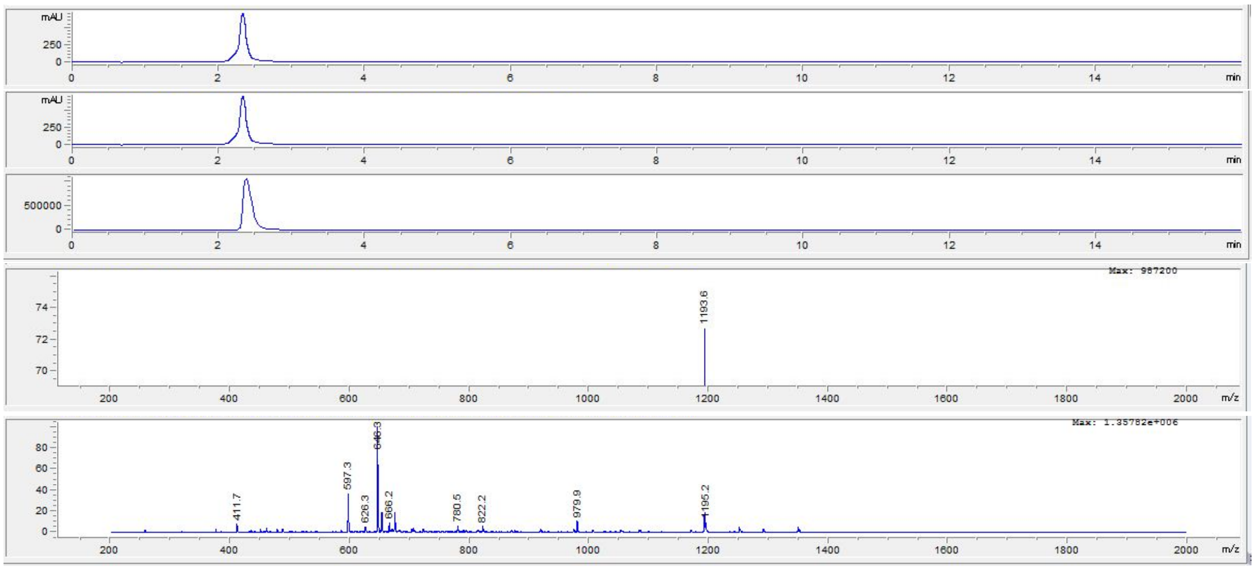

Chromatographic spectra at 280, 260, and 210 nm. Mass spectra at specific mass and total ion spectra.

Figure S4 - Structure and LC-MS Characterization of UreterGlow-11

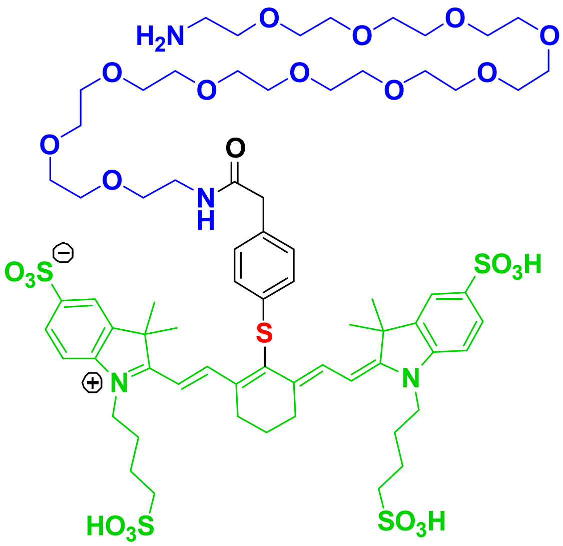

Chemical Formula: $C_{70}H_{104}N_4O_{24}S_5$
Molecular Weight: 1545.90600

UreterGlow-11

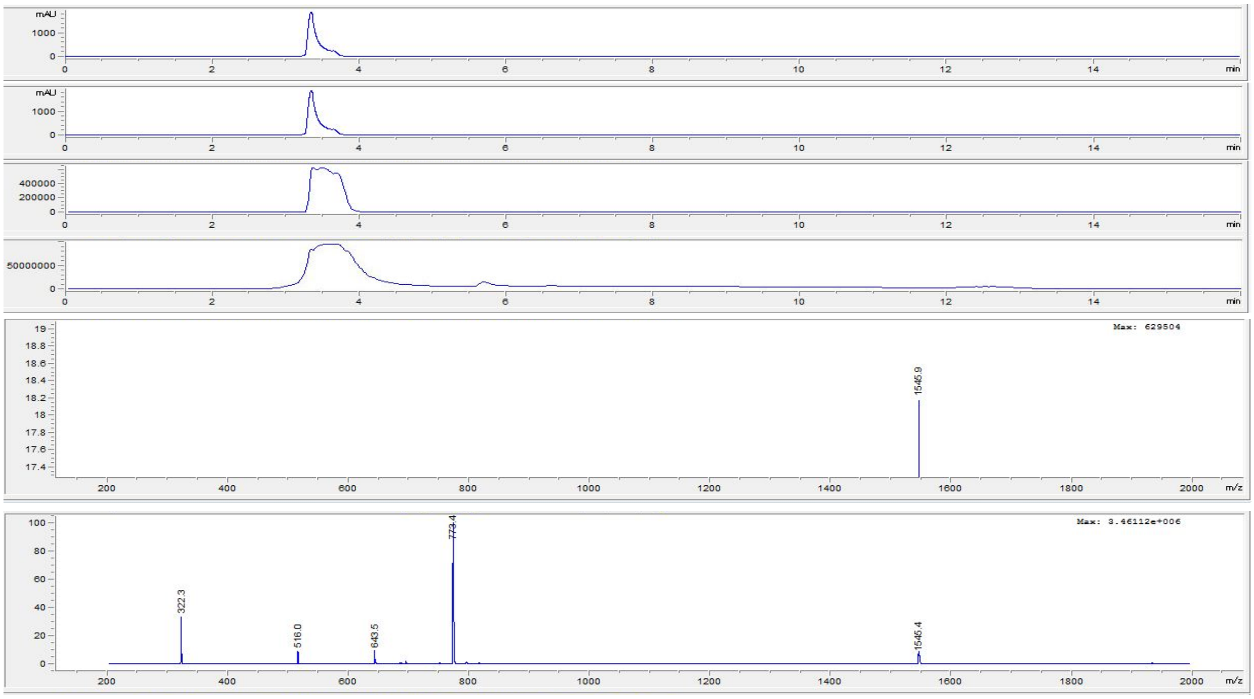

Chromatographic spectra at 280, 260, and 210 nm. Mass spectra at specific mass and total ion spectra.

Figure S5 - Structure and LC-MS Characterization of UreterGlow-45

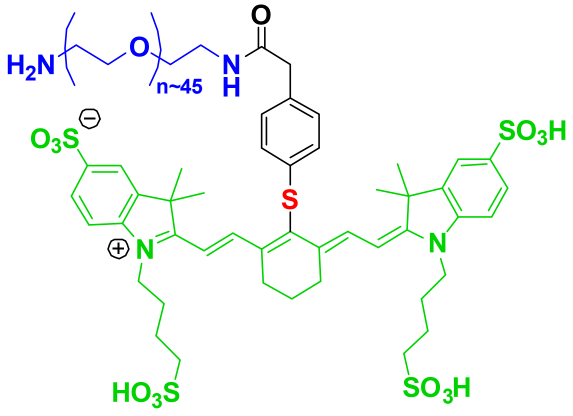

Chemical Formula: $C_{138}H_{240}N_4O_{58}S_5$
Molecular Weight: 3043.70800
UreterGlow-45

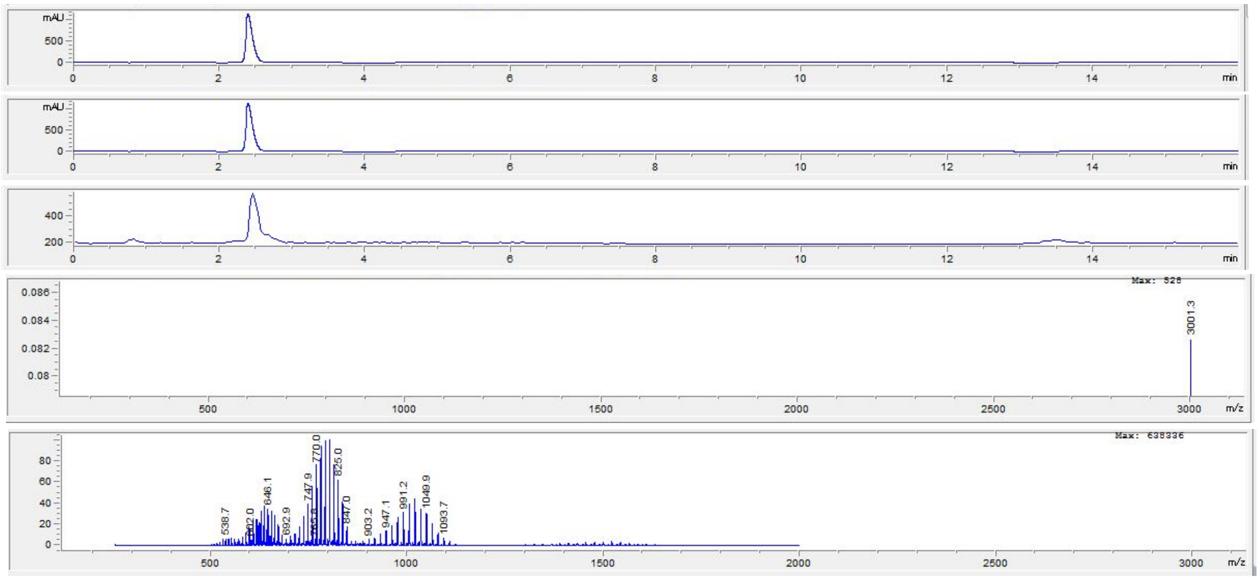

Chromatographic spectra at 280, 260, and 210 nm. Mass spectra at specific mass and total ion spectra.
